# Cardiac Amyloidosis: Tribulations and New Frontiers

**DOI:** 10.3390/jpm15100472

**Published:** 2025-10-02

**Authors:** Darren M. Nguyen, Noyan Ramazani, Gurpreet Sodhi, Tahir Tak

**Affiliations:** 1 Internal Medicine, University of Nevada Las Vegas, Kirk Kerkorian School of Medicine, Las Vegas, NV 89102, USA; 2 Advanced Heart Failure and Transplantation, Sutter Sacramento Medical Center, California Northstate University, School of Medicine, Elk Grove, CA 95758, USA; 3 Department of Cardiology, VA Southern Nevada Healthcare System, North Las Vegas, NV 89086, USA; tahir.tak@va.gov

**Keywords:** cardiac amyloidosis, restrictive cardiomyopathy, transthyretin, light chain amyloid, heart failure, tafamidis, acoramidis, diflunisal, inotersen, patisiran, vutrisiran, eplontersen, heart transplant

## Abstract

This review article aims to provide an overview of the pathophysiology, diagnosis, and contemporary management of cardiac amyloidosis (CA) as well as identify the knowledge gaps and areas of potential research. CA can be divided into two main groups: transthyretin cardiac amyloidosis (ATTR-CA) and light chain cardiac amyloidosis (AL-CA). The former further separates into wild-type transthyretin (ATTRwt) and hereditary transthyretin (ATTRv). African Americans, males, and people older than 75 are the most common demographics affected by this disease. Thanks to an increased understanding of this disease combined with better diagnostic techniques, there is growing awareness and a surge of clinical trials aimed at improving outcomes of CA. The diagnosis and treatment of CA is multifaceted and complex, relying on multiple imaging modalities and the cooperation of specialists to deliver effective treatments. While some disease-modifying agents have been introduced recently, their extraordinary cost limits their benefit or they are supported by limited evidence. Other agents are currently undergoing phase 3 trials. To date, there is scarce data surrounding optimal diagnostic and treatment strategies, including a potential role for combination therapies. Critically, it is imperative that physicians develop close relationships with the patient that addresses not only their individual health needs but also their unique psychosocial situation. Therefore, more clinical trials, protocols and patient resources are needed to better inform and guide providers managing these complex patient needs.

## 1. Introduction

Systemic amyloidosis is a progressive disease caused by infiltration of misfolded proteins, or amyloid fibrils, into various organ tissues, including the heart (cardiac amyloidosis).

There are two main forms of cardiac amyloidosis (CA): transthyretin cardiac amyloidosis (ATTR-CA) and light chain cardiac amyloidosis (AL-CA). ATTR-CA subdivides into wild-type (ATTRwt) and hereditary (ATTRv). CA is a significant cause of HF with preserved ejection fraction (HFpEF); ATTRwt is the etiology of 5% of these cases, which increases to 13% if left ventricular hypertrophy (LVH) is present [1,2]. In the United States, Medicare and Medicaid data show ATTR-CA increased in prevalence from 8 cases per 100,000 person years to 17 cases per 100,000 s persons from 2000–2012 [3]. Older people (>75 years of age), blacks, and males were the dominant demographic groups of ATTR-CA.

There are many possible mutations of the *TTR* gene that can cause ATTRv, with varying prevalence depending on the geographic region. In the United States, the Val112Ile mutation is the most common variant and usually found in males of African descent, seen in 3.4% of the population [4,5]. Other genotypes found in the United States are Thr60Ala, and Val50Met. Worldwide, Val50Met is the most common genotype with high prevalence in Europe, Japan, and South America [6]. Although Val50Met is the dominant genotype in Europe, some countries have their own prevalent genotypes, such as Leu111Met in Denmark, Ile68Leu in Italy, and Thr60Ala being the predominant genotype in both the United Kingdom and Ireland [7,8,9]. In China and Southeast Asian countries, Ala117Ser and Val50Met are the most prominent genotypes [10,11].

AL-CA is a manifestation of dysfunctional plasma cells that produce excessive amounts of unstable κ or λ light chains, which are deposited into a variety of organs including the heart [12]. While it shares similar pathophysiology to other plasma cell dyscrasia disorders, such as multiple myeloma, monoclonal gammopathy of undermined significance, and Waldenstrom macroglobulinemia, there is a difference in its pathophysiology. In AL-CA, the variable domain of the light chain can induce destabilization and misfolding of native proteins, allowing these proteins to form and propagate as amyloid fibrils [12]. In the United States, the prevalence of AL-CA increased from 22.7 cases per million to 69.1 cases per million with an annual growth rate of 32.1% over the period 2017–2021, according to Optum’s clinical database [13]. The people diagnosed tend to be older, a mean 67.2 years of age, white, and males [13]. In the United States, ethnic minorities tend to be underrepresented in such studies that calculate prevalence and incidence estimations due to healthcare disparities, lack of provider awareness, and other socioeconomic barriers [14]. Other countries have higher prevalence of AL-CA—there are 71.08 cases per million in Japan and 32.22 cases per million in Brazil [15].

**Figure 1:**
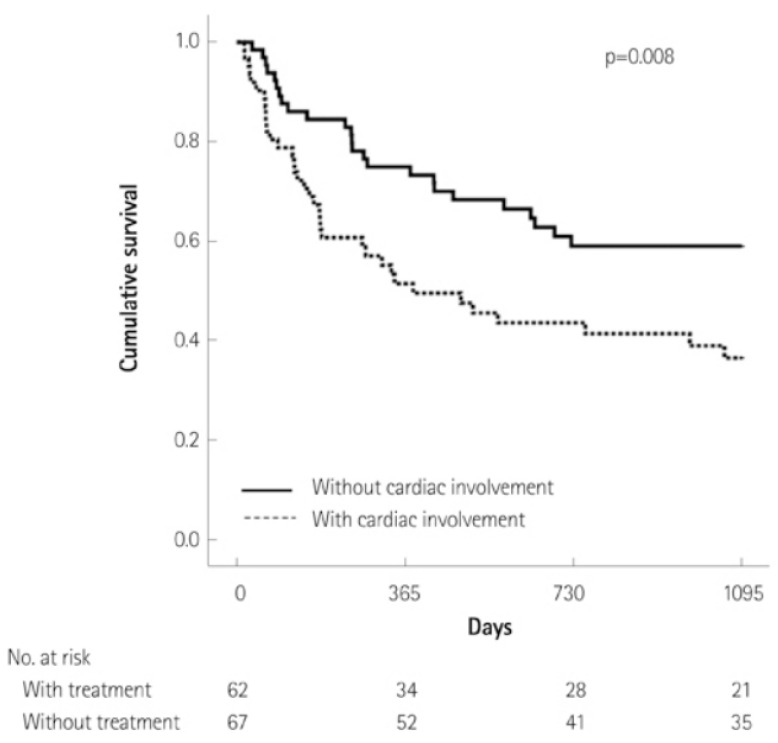
Kaplan–Meier curve of patients with amyloidosis with or without cardiac manifestations.

Systemic amyloidosis carries a high mortality rate, and cardiac involvement is the leading cause of death, as depicted in Figure 1 [16]. Escher et al. estimated patients with AL-CA have worse 5-year mortality rates compared to those with ATTRwt, with a 65% mortality rate in AL-CA compared to 44% in ATTRwt [17]. What contributes to the high mortality is the lack of tailored therapy and protocols available to treat people. While there are many FDA-approved medications for the treatment of amyloidosis, it is unclear in what ways and what situations they can be used due to the lack of data.

## 2. Clinical Presentation of Cardiac Amyloidosis

Amyloidosis is a systemic disease, affecting organ systems such as, but not limited to, the peripheral and autonomic nervous systems, gastrointestinal system (GI), and the kidneys [18,19]. Patients can initially present with mononeuropathy, such as carpal tunnel syndrome, years before the onset of cardiac symptoms [19]. Furthermore, amyloid infiltration of the GI tract can weaken the integrity of the mucosa and neuro-muscular network [20]. In advanced cases, amyloid patients can suffer with bleeding, ileus, gastroparesis, and alternating diarrhea and constipation [21,22,23]. Renal involvement is common, and microalbuminuria is an early sign of amyloid infiltration [24]. Half of these patients with microalbuminuria will progress to end-stage renal disease (ESRD) requiring dialysis [25].

Patients with CA have specific and non-specific HF symptoms that mimic ischemic and non-ischemic causes of HF [19]. Dyspnea on exertion, chest pain, palpitations, syncope, paroxysmal nocturnal dyspnea (PND), orthopnea, lower extremity edema, and abdominal ascites are some of the symptoms that CA patients may present with [19]. Arrhythmias are a life-threatening manifestation of CA with atrial fibrillation (AF) being the most common supraventricular arrhythmia, with a high incidence seen in ATTRwt, while ventricular arrhythmias can also occur in ATTR-CA, which puts patients at high risk of sudden cardiac arrest [26,27,28]. Other common features include signs of conduction disease, including AV block, intraventricular conduction delays, slow ventricular response, and even sudden cardiac death (SCD) [28].

**Figure 2:**
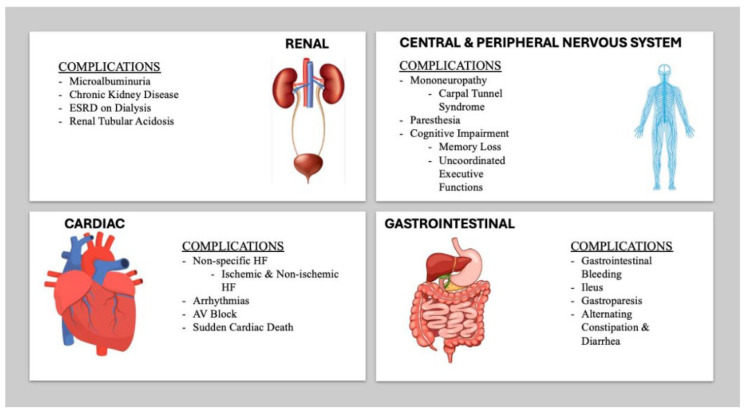
Clinical presentation of amyloidosis with negative implications for organ systems.

Contemporary consensus has identified certain “red flag” signs and symptoms to target providers’ pretest probability and increase opportunities for early diagnosis of CA [29]. These “red flag” signs and symptoms include, but are not limited to, symptoms of right HF, conduction abnormalities, autonomic neuropathy, and elevated cardiac biomarkers [29]. Identifying “red flags” with appropriate cardiac imaging, blood, and urine tests is the current accepted standard and moves patients one step closer to diagnosis. A summary of clinical manifestation of CA is shown in Figure 2.

## 3. Diagnosis of ATTR-CA

The limitations and logistical variables of each diagnostic modality can contribute to a delay in diagnosis. The differential diagnosis for HFpEF and non-ischemic cardiomyopathy (niCMY) are broad. Currently, there are limited recommendations for routine screening for ATTR-CA and AL-CA. This problem is a symptom of the lack of modern imaging modalities to specifically identify and target high-risk individuals, underscoring the need for protocols and guidelines to be developed that can specifically identify high-risk individuals and put them on the screening pathway. Doing so would prevent diagnostic delay and reduce frustration and medical costs for these individuals. The decision to pursue further workup is left to the clinician’s best judgement. When high suspicion prompts further evaluation of CA there are multiple imaging modalities that are effective at diagnosing CA and characterize the severity of the disease. Unfortunately, due to the rarity of the disease and its nonspecific clinical manifestations, patients with amyloid cardiomyopathy may have their diagnosis delayed or mistaken. Misdiagnosis occurs 34–57% of the time with an average 6.1 years of diagnostic delay for ATTRwt and 5.7 years for ATTRv [30]. Many of these patients report seeing more than two cardiologists before being diagnosed correctly [30]. Delays in diagnosis as well as multiple clinic visits without definitive answers can be frustrating to both patients and providers alike.

### 3.1. Overview of Diagnostic Modalities

**Figure 3:**
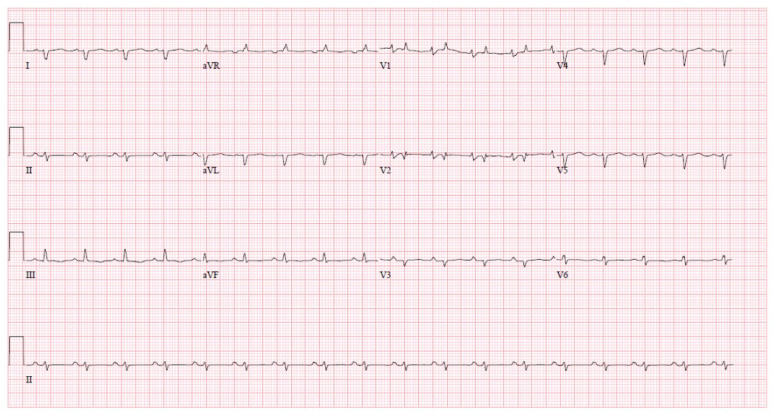
Electrocardiogram of a patient with amyloidosis with characteristic low-voltage findings in both the limb (QRS amplitude < 5 mm) and precordial leads (QRS amplitude < 10 mm). Used with permission from Dr. Gurpreet Sodhi.

The electrocardiogram (EKG) is one of the most ubiquitous diagnostic tools available to cardiologists. Some common EKG findings of CA include low voltage in limb leads, pseudo-infarct pattern, AV node blocks, and supraventricular arrhythmias such as AF [28,31]. Notably, low voltage, as shown in Figure 3, is present in 30% of patients with CA and is typically a sign of advanced disease [32].

**Figure 4:**
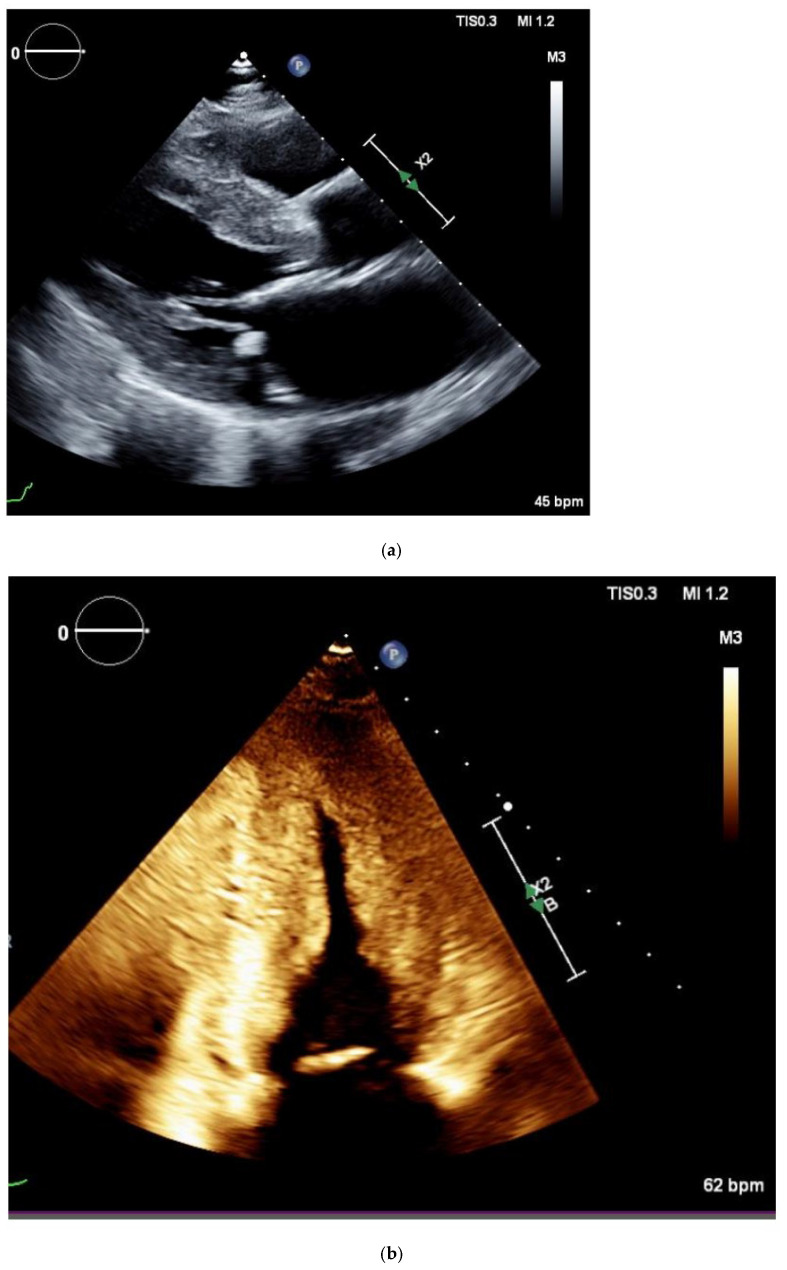
(**a**) Parasternal long axis echocardiogram view. Note the increased thickness of the interventricular septum. Used with permission from Sutter Sacramento Medical Center. (**b**) Echocardiography apical view of left ventricle. There is increased thickness of the interventricular septum and lateral left ventricle wall. Used with permission from Dr. Gurpreet Sodhi.

**Figure 5:**
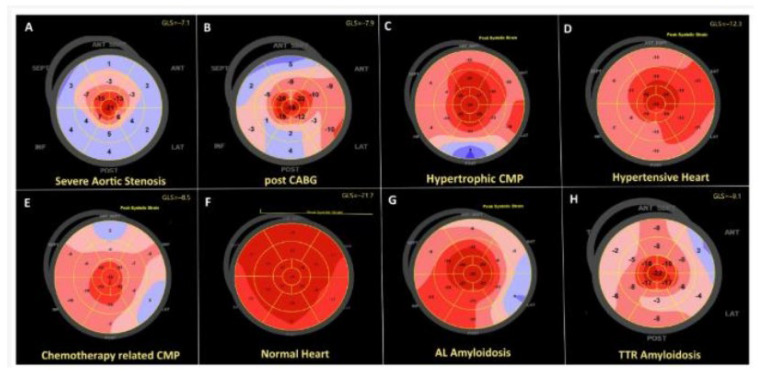
Speckle-tracking longitudinal strain patterns of different cardiomyopathies. Note that each pathology has apical sparing, as demonstrated by the dark red center of each image.

The echocardiogram is another important imaging modality for detection of CA. Findings suggestive of CA include LVH, diastolic dysfunction, and depressed left ventricular ejection fraction (LVEF) with findings depicted in Figure 4a,b [33]. The problem with the traditional LVEF measurement is that it lacks sensitivity to detect subclinical LV systolic dysfunction [34]. Global longitudinal strain (GLS) analysis with speckle tracking can detect subclinical cardiac dysfunction. It is a technique which analyzes the naturally occurring echocardiographic “speckles” within the myocardium to characterize LV strain [34]. Patients with amyloid cardiomyopathy are noted to have regional variations of longitudinal strain from base to apex; however, there is a relative apical sparing pattern [35]. This phenomenon is an accurate and reproducible method of differentiating cardiac amyloidosis from other causes of LVH, as depicted in Figure 5 [36]. Currently, clinical application of GLS is being explored in patients with CA. GLS is measured and reported as a proportion of the change of length of the myocardium compared to its baseline length; a higher delta change is indicative of healthy myocardium [37]. A GLS < −16% is abnormal, GLS −16 to −18% is borderline, and GLS > −18% is normal [37]. In other circumstances, it is typically used for tracking risk of chemotherapy-induced LV systolic dysfunction [37]. For CA, the measurement of the ratio of the EF to the GLS (EFSR) can be used to differentiate the etiology of LVH. A cutoff value of 4.1 or greater is used to predict amyloid infiltration as the cause of LVH at higher sensitivity and higher specificity than traditional echocardiographic markers [38].

### 3.2. Cardiac Magnetic Resonance Imaging

Cardiac magnetic resonance imaging (CMR) is an advanced imaging technique that analyzes the structure and function of the myocardium at any plane with no radiation required. In amyloidosis, the typical finding is global subendocardial late gadolinium enhancement, LVH, increased interatrial septal thickness, diastolic dysfunction, and increased cardiac mass [39]. Delayed enhancement-CMR (DE-CMR) is emerging as the optimal finding to diagnose CA. DE-CMR is performed by acquiring images 10–25 min after gadolinium contrast has been injected [40]. The underlying idea of this method is that there is a faster clearance of contrast in normal myocardium compared to abnormal myocardium. Myocardial nulling is an inversion recovery pulse sequence that “nulls” or darkens the normal myocardium while enhancing the abnormal myocardium [41]. This technique is helpful for identifying areas of myocardial scarring following an ischemic event, since infarcted myocardium has a slower washout time compared to normal myocardium [41]. Another feature in CA is the reversal of the nulling of the blood pool compared to the abnormal myocardium [41]. Normally, the blood pool should reach the nulling point before the myocardium; in CA, this process is reversed. Non-contrast T1 mapping is another technique for diagnosing CA [42,43]. Under the shortened modified look locker inversion recovery (ShMOLLI) sequence, images gathered from separate inversion recovery sequences are combined under a single dataset, all with a single breath hold for nine heart beats, making it more convenient for patients, especially those who cannot hold their breath for long [43].

**Figure 6:**
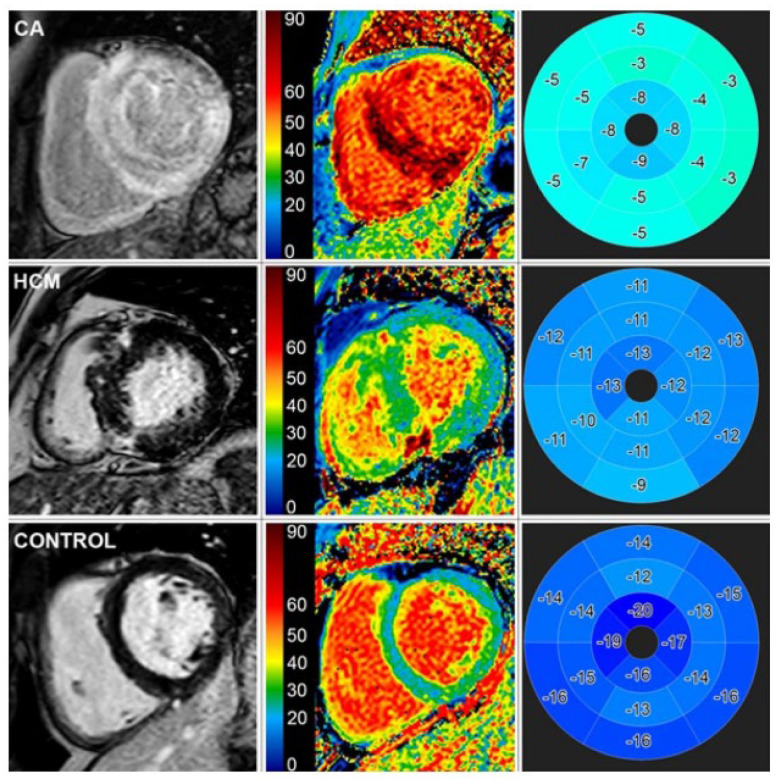
CMR images of cardiac amyloidosis. First column depicts late gadolinium enhancement; note the lack of enhancement of the myocardium in the CA group compared to the other two. Second column shows extracellular volume fraction; there are diffuse areas of elevated extracellular volume fraction. Third column contains speckle-tracking peak longitudinal strain; there is diffuse dyskinesis of the CA group compared to the others, as shown by the light blue/turquoise color and less negative values. CA: cardiac amyloidosis. HCM: hypertrophic cardiomyopathy.

In CMR, multiple parameters must be used to optimize the diagnostic sensitivity of the study. Extracellular volume (ECV) fraction measurement emerged as an important parameter to support the diagnosis of amyloidosis through CMR. ECV is calculated in contrast-enhanced T1 sequence mapping by measuring the myocardial relaxation times after contrast administration [44]. Normal values of ECV range from 25.3 +/− 3.5% [44]. Infiltration or scar within the myocardium will shorten T1 relaxation, which in turn will increase ECV [44]. T1 mapping and ECV (with cutoff of 46%) have excellent sensitivity in ATTR-CA and ECV can be an independent predictor of prognosis in these patients (a comparison is shown in Figure 6) [45,46]. However, in patients with prolonged T2 (>50 ms) as a result of inflammation or edema, the diagnostic accuracy of ECV declines [47].

T2 mapping is another method of not only evaluating the presence of amyloidosis but also differentiating the type. While native T2 values are elevated (mean 53.9 +/− 4.8 ms) in all types of CA, AL-CA had higher values compared to ATTR-CA [48,49]. The difference between the native T2 values could be a useful tool to discriminate the etiology of the amyloid disease.

### 3.3. 99-Technetium Pyrophosphate Imaging

**Figure 7:**
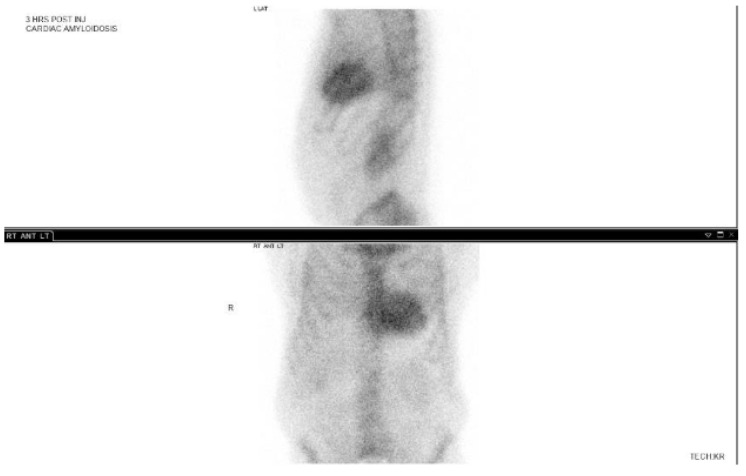
99tc-PYP scan of a patient with cardiac amyloidosis. There is an increased myocardium uptake. Used with permission from Dr. Gurpreet Sodhi.

99-Technetium pyrophosphate (Tc99-PYP) imaging is another effective imaging modality for ATTR-CA. This nuclear study involves administering a radiotracer ^99m^Technetium pyrophosphate, which has an affinity for calcium containing compounds. In other clinical settings, this test is used to characterize bone metabolism disorders or osteogenic bone malignancies. It is postulated that microcalcifications in amyloid proteins could be the cause of radiotracer uptake in the heart in the setting of amyloid cardiomyopathy [50]. Nevertheless, the degree of this radiotracer by myocardium is graded in accordance with the Perugini visual score. A grade 2 or 3 visual uptake is strongly suggestive of ATTR-CA, as shown in Figure 7. A recent study found that the 1 h protocol had similar sensitivity to the previous 3 h protocol [51]. If there is excess blood pool activity, a 3 h delayed scan should be performed to increase diagnostic accuracy [52,53]. A heart-to-contralateral lung ratio (H/CL ratio) can also be used to diagnose CA with a cutoff value of >1.5 being considered abnormal, and highly suggestive of CA [54]. Interestingly, subjects with ATTR-CA had higher H/CL ratios compared to subjects with AL-amyloidosis [54]. It is postulated that the characteristics of amyloidogenic fibrils in patients with ATTR cardiac amyloid may differ from those of AL amyloid, thereby resulting in higher levels of ^99m^Tc-PYP uptake. The exact mechanism by which the PYP scan can distinguish ATTR-CA versus AL-CA remains unclear [54].

### 3.4. Diagnosis of AL-Amyloidosis

In patients with suspected light chain amyloid disease, serum protein electrophoresis with immunofixation, urine protein electrophoresis with immunofixation, and serum-free light chain (FLC) ratio are recommended to evaluate light chain amyloidosis. The normal range of the serum FLC ratio is 0.26–1.65 [55]. It should be noted that patients with kidney disease and estimated glomerular filtration rate (eGFR) < 60 mL/min/1.73 m^2^ will have a serum FLC ratio of 0.37–3.10 [55]. Any FLC ratio above the reference range is considered abnormal. If all these tests are reassuring, then AL-CA is effectively ruled out [56].

An abnormal test does not necessarily diagnose AL-CA due to plasmacytosis or multiple myeloma without the clinical findings of cardiac amyloidosis or laboratory manifestations of it [51]. Rather, an abnormal test suggests a plasma cell dyscrasia that would require further testing or evaluation to ensure the patient does not have an underlying oncological process. Therefore, biopsy of the abdominal fat pad or of the affected organ site is required to diagnose AL-amyloidosis [57]. Tissue samples are then analyzed with mass spectrometry or immunohistochemistry to subtype the amyloidosis [57].

### 3.5. Role of Biopsy

A biopsy stained with Congo Red (apple-green birefringence under polarized light) is the gold standard for diagnosing amyloidosis [58]. According to the 2023 American College of Cardiology (ACC) expert consensus, there are two roles for biopsy, whether endomyocardial or extracardiac. Firstly, in patients with biopsy-proven AL-amyloidosis but with imaging findings suggestive of cardiac amyloidosis, it is reasonable, but not required, to pursue endomyocardial biopsy to rule out ATTRwt [59]. There have been numerous case studies documenting the presence of concurrent AL-amyloidosis with ATTRwt [60,61,62]. More commonly, AL-CA can be diagnosed through extracardiac tissue biopsy [63].

**Figure 8:**
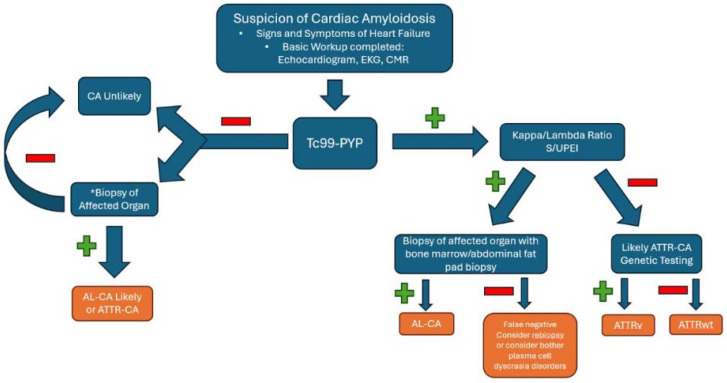
A diagnostic algorithm for cardiac amyloidosis. * Perform if suspicion of CA remains high despite negative 99-technetium pyrophosphate scintigraphy. AL-CA, light chain amyloidosis; ATTRv, hereditary transthyretin cardiac amyloidosis; ATTRwt, wild-type transthyretin cardiac amyloidosis; CA, cardiac amyloidosis; HFpEF, heart failure with preserved ejection fraction; Tc99-PYP, 99-technetium pyrophosphate scintigraphy. S/UPEI, serum and urine protein electrophoresis with immunofixation.

Secondly, patients with an abnormal monoclonal protein laboratory testing (either serum protein electrophoresis with immunofixation, urine protein electrophoresis with immunofixation, and serum FLC ratio) with strongly suspected cardiac amyloidosis, a biopsy is a reasonable next step to investigate [59]. The biopsy location should be considered as it can be from the abdominal fat pad, bone marrow, or the myocardium. A summary of the diagnostic algorithm for transthyretin cardiac amyloidosis is shown in Figure 8.

### 3.6. Discussion: A Comparison of Different Diagnostic Techniques

Each of the diagnostic techniques described above has strengths and weaknesses. While biopsy provides the definitive diagnosis for amyloidosis, it is only recommended in select situations given its invasiveness [59]. Prior to biopsy, patients often undergo more than one form of imaging before the diagnosis is made [56]. EKG and echocardiograms have the advantage of being quick, inexpensive, and readily available in a cardiologist’s office. However, the characteristic finding of low voltage is seen in only 30% of patients and is suggestive of late-stage disease [33]. Additionally, low voltage can be observed in other pathologies as well, such as cardiac tamponade, myocarditis, or chronic obstructive pulmonary disease (COPD) [64]. A finding of LVH in echocardiography is non-specific by itself as the LVH can be either due to hypertensive heart disease, renal disease, or other infiltrative cardiomyopathies. As discussed earlier, EFSR can be used to identify the etiology of LVH [38]. In a study comparing patients with biopsy proven CA and those with hypertrophic cardiomyopathy, traditional echocardiographic markers such as combining LV mass index with low-voltage EKG findings had a sensitivity of 35% and independently utilizing RV inferior wall thickening had a sensitivity of 25% [38]. Utilizing a cutoff of >4.1, employing an EFSR was shown to have a sensitivity of 89.7% for predicting cardiac amyloidosis [38]. Patients with CA had significantly higher EFSR compared to the LVH subgroups [38]. Overall, EFSR and GLS have emerged as promising parameters to not only workup LVH but also to risk-stratify these patients for survival. In patients with AL-CA and ATTR-CA with preserved EF, GLS can be used as an independent prognostic marker for survival [65,66]. In current practice, it is unclear which echocardiographic parameter—LV mass index, EFSR, or GLS—is the most accurate and should be used practically.

CMR is advantageous over echocardiography due to less dependence on operator skill and ability to generate high-resolution images at multiple different planes. CMR has a 87% diagnostic accuracy, which increases to 97% by combining presence of late gadolinium enhancement and T1 threshold between myocardium and blood based on a comparative study of 22 patients with biopsy-confirmed amyloidosis and 16 hypertensive controls [39]. Due to the use of gadolinium contrast, patients with a eGFR less than 30 mL/min/1.73 m^2^ are not candidates for contrast study [67]. Furthermore, removable or unremovable metallic implants and devices such as implantable cardiac defibrillators (ICD), pacemakers, and hearing aids are considered contraindications for CMR if these devices are not MRI-compatible [67].

Tc99-PYP imaging also carries excellent sensitivity and specificity at 97% and 100% respectively, using a H/CL >1.5 [54]. Unlike the other described imaging studies, Tc99-PYP has different results for ATTR-CA compared to AL-CA. Subjects with ATTR-CA had a higher absolute count in the heart region of interest compared to AL-CA; however, this finding is not statistically significant [54]. Patients with AL-CA can have a false positive on Tc99-PYP imaging because they too can have grade 2 or 3 myocardial radiotracer uptake, and similarly can have false negative rates [54]. If the AL-amyloidosis is excluded through negative urine/serum testing, then the positive predictive value of Tc99-PYP scans becomes 100% [54].

While biopsy is the gold-standard diagnosis, it is not without its flaws. For example, Quarta et al. found that abdominal fat pad aspiration had an 84% sensitivity for AL-amyloidosis, 45% sensitivity for ATTRv, and 15% sensitivity for ATTRwt [68]. Therefore, a negative fat pad biopsy does not eliminate transthyretin amyloidosis as the culprit of disease. To obtain the highest diagnostic yield, biopsies should be performed at sites of imaging abnormalities, and a bone marrow biopsy should be performed if there is a high pretest probability of plasma cell dyscrasia. Sometimes multiple biopsies involving different sites should be performed for the best chances of ultimately coming to a correct diagnosis of CA [69,70].

Fundamentally, there is no “best” non-invasive test for diagnosis of CA; often the diagnosis relies on multimodal imaging combined with laboratory testing and occasional biopsies. Nevertheless, a patient’s history and physical exam are a critical first step prior to testing and procedures. By understanding the patient’s individual history and clinical exam, the physician will be better able to tailor a diagnostic plan surrounding the patient’s circumstance to achieve an accurate diagnosis and better outcomes in a timely manner. However, this approach to personalized medicine is hampered by not only the rarity of the disease but also the lack of awareness among clinicians of the possibility of a rare disease presentation.

## 4. Management of Transthyretin Amyloidosis

A gap in current day guidelines is the role of TTR stabilizers and silencers in the management of heart failure. To date, there are no guidelines on the “optimal” regimen for CA. In current day practice, physicians would often use combination therapy of TTR-stabilizers and silencers as first line treatment for CA. However, it remains unknown whether traditional GDMT agents can be used along with TTR-specific mono or combination therapy. Moreover, there is a lack of data of long-term mortality, cardiovascular benefits of combination therapy with TTR-specific therapy with GDMT. Therefore, an element of personalized medicine comes into play that will test not only the knowledge of the physicians but also their relationship with and understanding of the patient. Before initiating treatment, the physician must understand their patient’s functional status from both a cardiovascular and neurological standpoint. Variables such as psychosocial support and financial situations must be considered as well. All of these factors together will influence the type of treatment regimen and even goals of therapy for the patient.

### 4.1. TTR Stabilizers

TTR stabilizers work by stabilizing the tetramer form of the TTR protein, which prevents dissociation and subsequent polymerization of the TTR fibrils. tafamidis is a newer medication approved in 2019 for the treatment of ATTRwt and ATTRv. Acoramidis recently completed its phase 3 trial in 2024. Other options include green tea extract and diflunisal.

### 4.2. Tafamidis

In a phase 3 multicentered, double-blind, placebo-controlled clinical trial, analyzing 411 patients with ATTRwt and ATTRv, tafamidis was shown to be superior to placebo in both of its primary end points: all-cause mortality and frequency of cardiovascular-related hospitalizations, with a win ratio of 1.695. tafamidis reduced all-cause mortality by 29.5% in the treatment group compared to 42.9% in the placebo group [71]. The study found that tafamidis’s relative risk of cardiovascular-related hospitalizations was 0.68 with patients having less of a decline in the 6 min walking distance test and improved quality of life [71]. Other studies also highlight the benefits of tafamidis, including that of Shah et al., which found that patients on tafamidis had less worsening of LV stroke volume, LV filling pressure, and LV global longitudinal strain over a 30-month treatment period compared to placebo [72]. Furthermore, Elliot et al. found that continuous dosing of tafamidis improved long-term survival [73]: 44.9% of patients in the continuous tafamidis group died, whereas 62.7% patients in the placebo group died after a 58-month follow-up [73]. The main drawback to tafamidis is its steep cost, with patients paying up to USD 225,000 per year for this treatment, significantly limiting access to this agent [74]. To alleviate the financial burden of the novel agent, Pfizer, the manufacturer of tafamidis, developed financial resource programs created in collaboration with third-party specialty pharmacies to help patients access this medication.

### 4.3. Acoramidis

Developed by AstraZeneca, this agent is the latest addition to the TTR stabilizer family. In a phase 3 double-blind placebo-controlled trial analyzing 611 patients with ATTRwt, acoramidis outperformed the placebo in all four of the study’s primary end points: death from any cause, cardiovascular-related hospitalization, change in NT-proBNP level, and change in baseline in 6 min walking distance [75]. In a four-step primary hierarchical analysis of the primary end points, acoramidis had a win ratio of 1.8 [75]. The relative risk of frequency of cardiovascular-related hospitalization was 0.496, favoring acoramidis [75]. In addition, patients in the acoramidis group had less decline in a 6 min walk test, with a least-squares mean difference of 39.6 m compared to patients in the placebo group [75]. Out of the four primary end points, the change in NT-proBNP heavily favored the acroamidis group, with 23.3% winners versus 7.0% losers [75]. The 30-month change of NT-proBNP was 0.529 in favor of acoramidis, meaning that patients saw half of the increase in NT-proBNP compared to the placebo group [75]. If approved for treatment of patients with ATTRwt, acoramidis would be a formidable competitor against tafamidis, especially if the former is affordable. However, the latter has the advantage of not only being in the market longer, but also has plentiful long-term studies compared to the former. Additionally, tafamidis surpasses its competitor in both reducing decline in 6 min walking distance (75.68 m vs. 39.6 m) and improved quality of life as measured by the Kansas City Cardiomyopathy Questionnaire–Overall Summary (KCCQ-OS) [71,75]. Compared to their respective placebos, tafamidis had a least-squares mean difference of 13.65 compared to acoramidis, which had a least-squares mean difference of 9.94 [75]. On the other hand, acoramidis reduced NT-proBNP in their treatment group, whereas tafamidis had an increase of NT-proBNP at 12 and 30 months into the trial [71,75]. More studies are needed to better understand the comparative efficacy of the two medications.

### 4.4. Diflunisal

Diflunisal is a non-steroidal anti-inflammatory drug (NSAID) that stabilizes transthyretin tetramers and prevents its misfolding monomers and dimers from forming amyloid deposits. In an observational study of 13 patients treated with diflunisal for one year, there were no significant differences in EF, interventricular septal thickness, and LV mass over the study period between baseline and post-treatment [76]. Overall, the study reported that diflunisal was well tolerated among the study group with minimal changes in platelets and GFR [76]. Nevertheless, caution is warranted when using diflunisal in the setting of kidney disease or peptic ulcer disease. Overall, diflunisal is not a generally accepted evidence-based treatment for amyloid disease despite its historic usage.

### 4.5. Green Tea Extract

Epigallocatechin-3-gallate (EGCG) is plentiful in green tea extract, and it inhibits fibril formation, thus hindering the propagation of transthyretin in in vitro experiments [77]. Similar to siflunisal, green tea extract’s limited data and lack of randomized control trials inhibit its acceptance as a viable treatment option. In an observational study, 19 patients with ATTR-CA were treated with green tea over the course of 12 months [78]. Of the 14 patients who completed the green tea regime, it was found that they had no increase in LV thickness [78]. In fact, CMR showed there was an average −12.5% decrease in myocardial mass in this group. Other reported findings included a significant decrease in total cholesterol and LDL [78].

### 4.6. TTR Silencers

TTR silencers, such as inotersen, patisiran, and vutrisiran, prevent hepatic synthesis of transthyretin through a variety of mechanisms. Patisiran is a siRNA, which binds onto TTR mRNA and tags it for degradation. Inotersen and eplontersen are antisense oligonucleotide (ASO) agents that work by binding onto TTR mRNA and marking it for degradation. Vutrisiran is a double-stranded siRNA-GalNAc conjugate that binds onto TTR mRNA, silencing it. Similar to tafamidis, patisiran, and inotersen are not readily accessible in pharmacies due to their steep costs. Patisiran is priced at USD 451,430–USD 677,145 per year depending on whether two or three vials are used for infusion [79]. Typically, two vials are given for those who weigh 34–66 kg, while three vials are given for those who are >66 kg. On the other hand, inotersen costs USD 420,000 per year [80]. Fortunately, there are specialty pharmacies that work with the respective drug companies and patients’ insurance companies to access the drug at affordable prices. Additionally, as mentioned, the drug companies have special programs targeting financial assistance and easier access to their respective silencer treatment. In terms of efficacy, a recent meta-analysis on TTR-specific therapy found that both silencer and stabilizer agents reduced all-cause mortality and improved KCCQ-OS and 6 min walk test (6MWT) distances [81], albeit no significant difference in terms of reduction of all-cause mortality was shown [81]. TTR-specific therapy was found to have no significant reduction in hospitalization or cardiovascular mortality compared to placebo. Nevertheless, a main limitation of this study is the demographic and clinical differences in patient cohorts used in the meta-analysis.

### 4.7. Inotersen

Inotersen is FDA-approved for ATTRv with polyneuropathy in 2018. In an international, multicentered double-blind placebo controlled study (n = 172), inotersen achieved significant changes in both of its primary end goals: improvement of both neuropathy and quality of life as ranked by the modified Neuropathy Impairment Score + 7 (mNIS + 7) and Norfolk Quality of Life Diabetic-Neuropathy (QOL-DN) questionnaire from baseline to 66 weeks of treatment [82]. In the inotersen group (n = 172), only 139 (81%) of patients completed the full 15-month regime [82]. Adverse reaction (n = 16, 14%) was the most common cause of stopping treatment early. Following completion of treatment, patients in the inotersen group had a +5.8 point in mNIS+ and +1 point in Norfolk QOL-DN questionnaire compared to their baseline [82]. In addition, inotersen fared significantly better compared to the placebo group. The difference in least-squares mean change from baseline to 66 weeks was −19.7 (*p* < 0.001) and −11.7 (*p* < 0.001) as measured by the mNIS + 7 and Norfolk QOL-DN questionnaire, respectively [82]. Long-term effects were studied with inotersen regarding improvement in neuropathy as a primary end point, but no studies to date have looked at cardiovascular outcomes on Inotersen [83].

### 4.8. Patisiran

Patisiran is not FDA-approved for treatment of ATTR-CA, but it is approved for treatment of polyneuropathy associated with ATTRv. APOLLO-B was a phase 3 double blind, randomized trial with a primary end point of change in distance of a 6 min walk test from baseline to 12 months of treatment [84]. One secondary end point was change in quality of life and functional status as measured by the KCCQ-OS [84]. There were 360 patients enrolled in this trial with 181 in the treatment group and 179 in the placebo group [84]. At the end of the 12-month treatment period, there was a significant difference in the primary end point between the two groups. The median change was −8.15 m and −21.35 m in the patisiran and placebo groups, respectively [84]. In addition, the placebo group had a 3.4 KCCQ-OS point decline after 12 months compared to baseline, whereas the patisiran group had a 0.3 increase in KCCQ-OS points after 12 months [84]. As mentioned earlier, the results of the APOLLO-B trial were not sufficient to prove the efficacy of patisiran for ATTR-CA. In October 2023, according to a complete response letter from the FDA, the organization notes that while no “clinical safety, study conduct, drug quality, or manufacturing issues” could be identified, the TTR silencer lacked evidence of “clinical meaningfulness” [85]. Alnylam, the manufacturer for the agent, announced that it will not pursue an expanded indication of patisiran in the United States [85]. However, the agent remains available to patients enrolled in the open label extension period of the APOLLO-B trial and patisiran expanded access protocol [85]. Given its novelty, there is not a long-term study looking at improvement in cardiovascular function as a primary end point.

### 4.9. Vutrisiran

With patisiran failing to meet FDA requirements, vutrisiran remains Alnylam’s other chance at entering the ATTR-CA market. Currently, this agent is being evaluated in the HELIOS- A and HELIOS-B Trial, which is a phase 3, randomized, placebo-controlled, double-blind, multicenter study. HELIOS-A looks specifically at vutrisiran’s efficacy for ATTRv while HELIOS-B evaluates the medication’s efficacy for ATTRwt [86,87]. The latter enrolled 655 patients and is estimated to be completed in 2026 [87].

### 4.10. Eplontersen

Eplontersen is another ASO, like inotersen. It was recently approved by the FDA in 2023 for patients with ATTRv with polyneuropathy [88]. In its phase 3 international trial (NEURO-TTRansform), eplontersen outperformed placebo in all the primary end points [89]. Over a period of 66 weeks, there was a 0.3 mean change in baseline mNIS + 7 scores in the treatment group compared to a 25.1 mean change in the placebo group, indicating less worsening of baseline neuropathy in the intervention group [89]. Furthermore, there was a −5.5 adjusted mean change in Norfolk QoL-DN score in the eplontersen group as opposed to a 14.2 adjusted mean change in the same score for the placebo group [89]. This difference demonstrates that patients on eplontersen have improved quality of life while on the intervention [89]. At the end of the 66-week treatment period, the serum transthyretin count was reduced by 81.7% in the treatment group [89]. While there is no approved indication for ATTR-CA, this novel agent is currently being evaluated in the CARDIO-TTRansform trial to establish its efficacy for patients with ATTR-CA.

### 4.11. Monoclonal Antibodies

Monoclonal antibodies are emerging to become a new, innovative tool in combating amyloid disease. PRX004 and NC1006 are two agents in phase 1 trials. PRX004 binds onto amyloid fibrils, promoting antibody-mediated phagocytosis [90], whereas NC1006 aids in removing amyloid fibrils from the heart [91]. Though it is in its earliest stages, NC1006 has shown potential in its phase 1 clinical trial. Researchers found no serious adverse effects from administration of NC1006 [92]. Because of this untapped potential, more trials should be undertaken to evaluate the efficacy and safety of monoclonal antibodies for patients with ATTRwt and ATTRv.

### 4.12. Gene Editing

**Figure 9:**
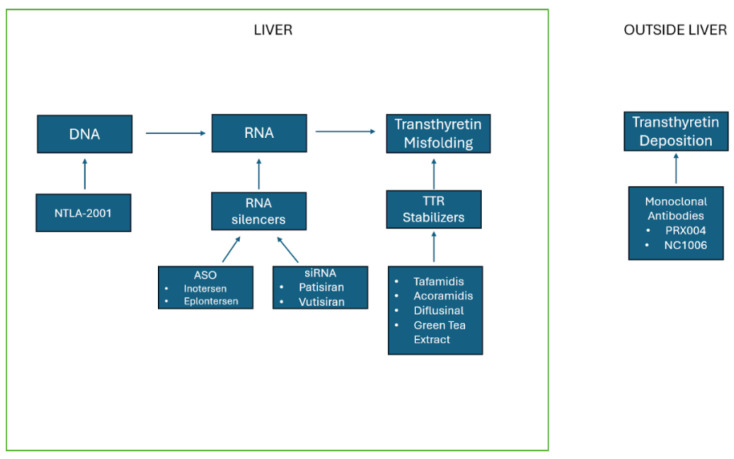
Current treatment for transthyretin cardiac amyloidosis on a molecular level. ASO, antisense oligonucleotide; TTR, transthyretin.

Gene editing represents the best avenue towards a true individualized approach to CA. By identifying a faulty gene or genes in the individuals, a specific agent can be selected to rectify that abnormality. One developing agent, nexiguran ziclumeran (nex-z) or NTLA-2001, developed by Intellia Therapeutics, is a CRISPR-Cas9 lipid nanoparticle (LNP) delivery system with a single guided RNA (sgRNA) that targets the *TTR* gene in hepatocytes to reduce transthyretin concentrations in serum. In mice models, a single dose of nex-z reduced serum TTR levels by >97% persisting for more than 12 months [93]. Given the success in animal models, there is untapped potential for benefit in humans. In its phase 1 study, nex-z has been shown to cause few side effects in patients with hereditary ATTRv with polyneuropathy [94]. The safety profile of nex-z is also validated in patients with ATTRwt. Some patients experienced infusion-related reactions such as fevers, headaches, back pain, and hypotension, which were treated with acetaminophen and intravenous fluids [95]. In both studies, there was a significant decrease in serum TTR concentration as well as decrease in NT-proBNP [96,97]. Patients on nex-z were found to have no worsening of their ATTR-CA disease at 12 months as measured by NT-proBNP, troponin T or 6 min walk test, and a median increase of 8 points on their KCCQ score [95]. A criticism of CRISPR-Cas9 is the potential for off-target gene editing leading to undesired results [96]. The LNP delivery system has a binding affinity for the liver because it contains apolipoprotein E (ApoE) [97]. ApoE is readily recognized by low-density lipoprotein (LDL) receptors and absorbed into the liver [98]. Currently, NTLA-2001 is being evaluated in the MAGNITUDE trial, a phase 3 study of patients with ATTR-CA with expected completion in 2028 [99]. A summary of the current and novel treatments of ATTR-CA is shown in Figure 9.

## 5. Medical Management of Heart Failure

The most recent guidelines for HFpEF strongly recommend the use of loop diuretics such as furosemide to alleviate congestion and improve symptoms in combination with certain guideline-directed medical therapy (GDMT) [100]. As a first line agent, sodium-glucose cotransporter-2 inhibitors (SGLT-2i) are also recommended by the guidelines [100]. This recommendation is based on the EMPEROR-PRESERVED and DELIVER trials, which showed reduction in HF hospitalizations and cardiovascular deaths with empagliflozin and dapagliflozin respectively [101,102]. Initiation of angiotensin receptor blockers (ARB), angiotensin-receptor neprilysin inhibitors (ARNI) as well as mineralocorticoid receptor antagonists (MRA) are all recommended [100]. Recently, finenerone was shown in its phase III trial to reduce hospitalization due to HF and reduce cardiovascular deaths for patients with HF with mildly reduced ejection fraction (HFmrEF) as well as HFpEF [100]. It should be noted that the data for the use of SGLT-2i and finenerone in patients with CA is limited. For example, the DELIVER, EMPEROR-Preserved, and FINEARTS trials all excluded patients with CA and other infiltrative cardiomyopathies [101,102,103]. Therefore, future studies should aim to analyze clinical and mortality benefits of these treatments for patients with CA and other infiltrative cardiomyopathies. Other traditional GDMT, such as ARB, ARNI, and beta blockers should be used with caution in this patient population due to risk of hypotension; extra caution should be taken for the patients with autonomic dysfunction [59,103].

Transthyretin modifying therapy such as tafamidis is recommended in all patients with CA. Tafamidis is indicated in patients with New York Heart Association (NYHA) Class I to III symptoms [59,100,104]. The ongoing trials of the TTR-Inhibitor and recent completion of phase III trial of acoramidis present new opportunities for treatment strategies for CA.

Tachyarrhythmias presents a major complication of ATTR-CA. As stated previously, AF is the most common arrhythmia in patients with cardiac amyloidosis [27,28]. Beta blockers and non-dihydropyridine calcium channel blockers should be used with caution as patients rely on heart rate to maintain adequate cardiac output in the setting of a fixed stroke volume [104]. In AL-CA, non-dihydropyridine calcium channel blockers can bind onto amyloid fibrils, leading to decompensated HF or shock [104]. Digoxin was once contraindicated in CA because it was demonstrated to bind onto amyloid fibrils; however, recent expert consensus concluded that digoxin can be used for rate control albeit with close monitoring [59,104]. There is limited data on rhythm control in patients with CA with AF. While amiodarone is well tolerated at low doses, it does not provide a mortality benefit [105]. Multiple retrospective and observational trials support the efficacy of rhythm control strategies such as amiodarone, cardioversion, and catheter ablation over rate control, especially when used early in the disease course [105,106,107]. Despite that, there are no recommendations for rhythm control over rate control or vice versa in the recent guidelines [59,104].

Current HF guidelines and expert consensus both agree that anticoagulation is always necessary, unless contraindicated, for AF in the setting of CA, regardless of CHA_2_DS_2_VASC score [59,100,108]. This recommendation is made based on a retrospective cohort study of 100 patients with AF and CA, who received a transesophageal echocardiogram. In that cohort of patients, 30% had a left atrial appendage (LAA) thrombus identified [109]. The researchers found that “the incidence of LAA thrombus did not demonstrate a proportional increase in relation to CHA_2_DS_2_VASC score” [109]. There are no formal recommendations on direct oral anticoagulation (DOAC) versus warfarin in this specific patient population. In contemporary practice, DOAC is preferred given its ease of usage, dosage, and monitoring [59]. In patients with contraindications to anticoagulation, LAA closure remains the last option for these patients [59].

## 6. Device Therapy

Ventricular arrhythmias can occur in CA, which contributes to half of SCD events. SCD usually occurs because of electromechanical dissociation, which can lead to pulseless electrical activity (PEA). The data on survival benefits with ICD implantation for primary or secondary prevention is limited and conflicting. While there is evidence that ICD can terminate life-threatening ventricular arrhythmias, it is not associated with improved mortality [110,111,112]. With the limited data and studies available, in 2017 the American Heart Association/American College of Cardiology/Heart Rhythm Society recommended individualized decision-making pertaining to ICD placement with patients with CA [113].

The utility of permanent pacemakers (PPM) has been explored for management of brady arrhythmias such as AV or sinus nodal dysfunction secondary to amyloid disease [1]. The most common indication for PPM is complete AV nodal block [114,115]. Other indications include atrial fibrillation, type 1 AV block, and bifascicular block [115,116]. Among the CA variants, ATTRwt is the most likely to have a PPM. The need for a pacemaker in a patient with CA indicates advanced disease, carrying a poor prognosis [115]. Having PPM does not improve outcomes, as these patients are at higher risk of all-cause mortality (hazard ratio = 1.58), worsening heart failure symptoms, and higher rates of hospitalization [115].

In the presence of prolonged QRS either due to left bundle branch block (LBBB) or right bundle branch block (RBBB), the benefits of cardiac resynchronization therapy (CRT) has been investigated. In a small trial of 30 participants with heart failure (EF < 30%) and LBBB or RBBB, CRT was found to have superior mortality and symptomatic relief compared to medical therapy alone [117]. By contrast, in a similar population group, benefits of CRT were compared to those with dilated cardiomyopathy [118]. At the end of a 2-year follow-up, CA patients with CRT had worse echocardiographic improvement in heart failure when compared to those with CRT and dilated cardiomyopathy. There were also worse outcomes in terms of all-cause mortality and major adverse cardiac events (MACE) in the CA cohort [118]. As such, the role of CRT in patients with CA is controversial. While the aforementioned studies evaluated the efficacy of CRT in ATTR-CA, patients with AL-CA have not been widely studied [117]. Nevertheless, CRT can be beneficial in niche cases that meet conventional guideline indications [117].

## 7. Management of AL-Amyloidosis: Chemotherapy and Stem Cell Transplantation

Patients with AL-CA need to be evaluated to confirm whether they are candidates for autologous stem cell transplantation (ASCT). If qualified, patients who undergo ASCT have fair long-term survival rates. Cordes et al. reported a 43% 10-year survival rate [119]. Extending the timeline further, Sidana et al. reported a 30% 15-year survival rate [120]. Factors that affect survival are the number of organs involved, and the types of organs involved [119]. Additionally, total cholesterol and total urine protein were also factors [119]. Cardiac involvement was found to depress survival rates in both studies [119,120]. It should be noted that patients with ESRD, in both criteria, are not automatically excluded if they meet the other criteria and are on dialysis [119,120]. Historically, patients who are eligible for ASCT and have bone marrow plasmacytosis > 10% should undergo two to four cycles of induction therapy with a bortezomib, cyclophosphamide, and dexamethasone (CyBorD) [121]. Recently, the ANDROMEDA trial found that the addition of daratumumab to CyBorD showed a better hematologic response and less disease progression [122]. Following this landmark trial, the standard of care is daratumumab with CyBorD (Dara-CyBorD) in patients who are either ASCT candidates or not [121,123]. Of note, if complete remission is achieved, then ASCT can be deferred [121]. Prior to ASCT, conditioning therapy is required to eliminate aberrant plasma cells and create room for new stem cells to grow and proliferate. Typically, high-dose melphalan is used as conditioning therapy. In 40% of patients, remission is achieved after ASCT and melphalan and these patients have improved survival [121,123]. Current guidelines do not recommend maintenance chemotherapy [121]. Novel AL-CA treatments such as venetoclax, which is a BCL2 inhibitor, belantamab-mafodotin, which is a B-cell maturation antigen inhibitor, and selinexor, which inhibits Exportin1, are being currently investigated [124].

## 8. Heart Transplantation

Heart transplantation (HT) is the final step of the amyloidosis treatment algorithm. There are numerous centers in the United States that offer heart transplantation. While many of these centers have differing criteria as to who can qualify for transplantation, in general, patients who have severe heart disease unresponsive to maximal medical therapy are typically considered as candidates [125]. Historically, those with ATTRwt can be considered for HT; patients with ATTRv ought to be considered for combined heart and liver transplantation as a way to halt the progression of neuropathy [59,125]. In most cases, patients who meet criteria for transplantation will usually receive heart transplantation alone. Patients with AL-CM can be transplant candidates if they are not candidates for disease-specific therapy. For this population, following HT, it is recommended that ASCT be performed as well if they are candidates [125]. Transplant guidelines recommend additional screening for extra-cardiac manifestations of amyloidosis (pulmonary, GI, hepatic, or renal) [125].

Thanks to advances in immunosuppression and post-transplantation care, there is improved survival after transplantation and there is less risk of rejection within 1 year [126]. The median survival for post-transplant patients is 10.7 years based on a multinational registry that tracks heart transplantation from 1982 to 2015 [126]. Compared to patients who received heart transplantation for any other reason, patients who received heart transplantation for CA have similar risk of bleeding, infection, renal failure, rejection or malignancy [127]. Furthermore, there is no significant difference in mortality between the two groups [127]. Overall, heart transplantation remains a viable option for patients with CA refractory to mechanical circulatory support or maximal medical therapy.

## 9. Importance of Multidisciplinary Approach

Because of the multi-organ involvement of AL-CA and ATTR-CA, care by a single specialty is insufficient to optimize care for the patient. For ATTRv, while there is no standard guideline for screening and treatment for high-risk asymptomatic patients such as first degree relatives of these patients, experts agree that early detection combined with routine monitoring of asymptomatic individuals is beneficial for their overall outcomes [128]. Thanks to the development of new agents, early treatment of these individuals can also improve outcomes [129]. While disease-modifying therapy is effective at hindering the progression of amyloid disease, involving other specialties to provide a multidisciplinary approach to these patients is beneficial in addressing the variety of complications and challenges posed in CA. For example, patients with severe neuropathic pain may need to be referred to neurology or anesthesiology for further treatment [130]. Likewise, patients who suffer from malnutrition and intestinal obstruction will benefit from a GI specialist with nutritionist support [59].

For patients with AL-CA, hematologists can play a crucial role in the management of these patients. Firstly, they can interpret abnormal monoclonal protein screens and further evaluate patients with abnormal screens. Subsequently, they can initiate chemotherapy and evaluate patients for stem cell transplantation. Progressive renal dysfunction can occur in amyloidosis; nephrology should be involved in addressing the kidney-related complications of light chain amyloidosis such as nephrotic syndrome, cardiorenal syndrome, and metabolic abnormalities. To mitigate these complications, nephrologists can assist in initiating renal replacement therapy as well as providing input into possible renal transplant. Renal transplantation has better outcomes compared to dialysis. In patients with AL-amyloidosis with renal manifestations, the median survival was 39 months on dialysis in a United Kingdom study [131], whereas patients with light chain nephropathy who received kidney transplantation had a median survival of 8.6 years [132]. Patients with complete hematologic response or significant partial response to chemotherapy prior to transplantation had better outcomes post-transplantation [133]. The CA patient is a complex picture, with multiple organ manifestations and interwoven problems; a single physician could be overwhelmed. This scenario underscores the importance of a multidisciplinary approach to these patients to improve patient care and outcomes.

Lastly, the importance of the patient–physician relationship cannot be neglected. As amyloidosis is a chronic disease, certain academic centers and private hospital groups have formed programs specializing in the long-term treatment of amyloidosis. While many of these centers have access to the latest treatment and the best specialists, it is important for the individual physician to know their patient well from both a medical and social perspective. Topics such as their neurological status, cardiovascular status, frequency of hospitalization and transplant candidacy are important. On the other hand, knowing their family life, their financial status, employment, and outside obligations are equally important as well. To address and overcome present and potential barriers, physicians ought to be comprehensive and systematic in their approach with working these patients with their own unique situations. Offering patient resources such as reduced co-pay on specialized treatment, transportation, networking between specialists, and home-health support, patient liaisons are all critical to a comprehensive treatment of CA. Additionally, Sutter offers patient support groups in which patients with amyloidosis will meet and discuss their experiences. Akin to group therapy sessions commonly employed in psychiatric practice, these support groups allow patients to bond and relate through shared experience and can even be therapeutic as well. Depression and anxiety are common symptoms of chronic disease, both of which can be debilitating. By giving the patients the right resources and opportunities, they can be empowered to find their own ways to live with this chronic illness by being better informed and motivated.

The mode and strategy of treatment ought to be tailored to the patient’s individualized circumstances and socioeconomic status. As systemic amyloidosis is a chronic progressive condition, it is important for physicians to build and maintain a trusting relationship with the afflicted patient to aid and alleviate the complications of the natural history of amyloidosis.

## 10. Future Directions

Diagnosis and treatment of CA are the two main challenges in managing CA. There is a critical lack of data looking at optimal diagnosing and treatment of CA. Guidelines have been equivocal when addressing this topic owing to the lack of data. Fortunately, trials are under way to explore novel options for diagnosing CA. For example, artificial intelligence (AI) is being used to enhance diagnostic accuracy of both EKG and echocardiograms [134,135]. AI can be taught how to recognize pertinent image findings and support clinical decision-making. A computer-aided diagnosis (CAD-Dx) was used during a colonoscopy had a higher sensitivity of 90.4% compared to visualization, which had a sensitivity of 88.4% [136]. A deterioration index (DI) is an early-warning system used by AI integrated into the electronic medical record to improve risk stratification for patients [137]. Using a score of >60, the index had a sensitivity of 88.5% and a specificity of 59.8% [137]. AI shows promise in resolving the diagnostic conundrum that is prevalent in CA.

In addition to improving diagnostic techniques for CA, more treatments are being tested that can revolutionize the way CA is treated. Established TTR silencers such as eplontersen and vutrisiran are being tested on patients with ATTR-CA in the CARDIO-TTRansform and HELIOS-B trials, respectively. Novel monoclonal antibodies, while in phase 1 trials, show strong potential to reverse the effects of amyloid deposition in the heart [90,91]. A brief selection of ongoing diagnostic studies and clinical trials is shown in Table 1.

## 11. Conclusions

At times, CA remains an elusive diagnosis due to several non-specific clinical symptoms and inherent rarity, leading to a low upfront clinical suspicion at time of patient presentations. These variables often confuse clinicians, causing delay in the diagnosis or misdiagnoses, and initiation of unnecessary medical interventions, leading to frustration for both patients and physicians. While there are treatments for CA, their costs limit their readily accessibility, but fortunately special programs through drug companies connected with specialty pharmacies make it easier for patients. Even with the right resources in place, there remains a lack of precision medicine when it comes to the overall treatment of these people. Lack of data in medication regimens and tailored diagnostic protocols represent two major challenges in this subject. Nevertheless, awareness of CA continues to improve, and personalized medicine is gaining popularity among clinicians of all fields. To strengthen treatment and provide better outcomes, more clinical research pertaining to diagnostic selection and treatment strategies should be carried out.

## Figures and Tables

**Table 1:** Selection of important ongoing clinical trials for the management and diagnosis of cardiac amyloidosis.

Study Title	Intervention	Number of Study Participants	Study Progress
**HELIOS-A** [79]	Efficacy of vutrisiran for hereditary transthyretin amyloid cardiomyopathy	164	Estimated Completion: 10/2026
**HELIOS-B** [80]	Efficacy of vutrisiran for transthyretin amyloid cardiomyopathy	655	Estimated Completion: 12/2026
**CARDIO-TTRANSFORM** [126]	Efficacy of eplontersen for transthyretin amyloid cardiomyopathy	1438	Estimated Completion: 11/2025
**MAGNITUDE** [92]	NTLA-2001 transthyretin amyloid cardiomyopathy	765	Estimated Completion: 4/2028
**Early Detection of Neuropathy in ATTRv** [127]	Comparing different diagnostic modalities for early diagnosis of peripheral neuropathy in patients with ATTRv	47	Estimated Completion: 2/2029
**Exploring Biomarkers in Hereditary Amyloidosis** [128]	Characterizing different biomarkers to better characterize severity and natural course of amyloid disease	80	Estimated Completion: 4/2026
**Artificial Intelligence Enhanced ECG to Detect Cardiac Amyloidosis** [129]	Utilization of artificial intelligence to improve diagnosis of amyloidosis	200	Estimated Completion: 12/2024
**Artificial Intelligence Guided Echocardiographic Screening of Rare Diseases (EchoNet-Screening)** [130]	Utilization of AI to identify patients with LVH. AI will determine whether these patients need additional screening	300	Estimated Completion: 6/2025

## Data Availability

No new data were created or analyzed in this study. Data sharing is not applicable to this article.
